# Neuroactive compounds obtained from arthropod venoms as new therapeutic platforms for the treatment of neurological disorders

**DOI:** 10.1186/s40409-015-0031-x

**Published:** 2015-08-08

**Authors:** Victoria Monge-Fuentes, Flávia Maria Medeiros Gomes, Gabriel Avohay Alves Campos, Juliana de Castro Silva, Andréia Mayer Biolchi, Lilian Carneiro dos Anjos, Jacqueline Coimbra Gonçalves, Kamila Soares Lopes, Márcia Renata Mortari

**Affiliations:** Laboratory of Neuropharmacology, Department of Physiological Sciences, Institute of Biological Sciences, University of Brasília, DF CEP 70.910-900, Brasília, Brazil

## Abstract

The impact of neurological disorders in society is growing with alarming estimations for an incidence increase in the next decades. These disorders are generally chronic and can affect individuals early during productive life, imposing real limitations on the performance of their social roles. Patients can have their independence, autonomy, freedom, self-image, and self-confidence affected. In spite of their availability, drugs for the treatment of these disorders are commonly associated with side effects, which can vary in frequency and severity. Currently, no effective cure is known. Nowadays, the biopharmaceutical research community widely recognizes arthropod venoms as a rich source of bioactive compounds, providing a plethora of possibilities for the discovery of new neuroactive compounds, opening up novel and attractive opportunities in this field. Several identified molecules with a neuropharmacological profile can act in the central nervous system on different neuronal targets, rendering them useful tools for the study of neurological disorders. In this context, this review aims to describe the current main compounds extracted from arthropod venoms for the treatment of five major existing neurological disorders: stroke, Alzheimer’s disease, epilepsy, Parkinson’s disease, and pathological anxiety.

## Introduction

Important ecological advantages are conferred to a diverse range of animals that evolved a finely tuned venom system adapted for predation, defense, and competitor deterrence purposes. From the evolutionary point of view, the prey–predator relationship applies constant selection pressure on toxin efficacy by demanding high specificity and potency for their molecular targets, primarily in the cardiovascular and nervous systems. These features are not commonly found in other natural or synthetic small molecules, making animal toxins extremely valuable as pharmacological tools [1, 2]. Nowadays, many major pharmaceutical companies develop venom-based drug discovery programs or use venom-derived molecules for target validation. Furthermore, there are now several companies focusing on venom-derived therapeutics [2].

A series of venom-derived peptides and proteins are currently in preclinical development while some others are undergoing clinical trials for their applications in the treatment of cancer, chronic pain, congestive heart failure, epilepsy, myocardial infarction, stroke, and multiple sclerosis [1–3]. Six medications obtained from venom proteins and derivatives – namely captopril (Capoten®), eptifibatide (Integrilin®), tirofiban (Aggrastat®), bivalirudin (Angiomax®), ziconitide (Prialt®), and exenatide (Byetta®) – have been approved by the U.S. Food and Drug Administration (FDA), targeting hypertension, acute coronary syndromes, coagulation during surgery, chronic pain, and type 2 diabetes [3]. Nevertheless, novel technologies (*i.e*.: proteomics approach) have become key components for bioprospecting, unveiling new molecular components in venoms that provide blueprints to treat a wider variety of disorders, such as neurological diseases [4].

Neurological disorders, that encompass, among others, both neurodegenerative and neuropsychiatric conditions, have attracted great interest due to their high impact on society [5]. Most of these disorders have a chronic profile, presenting socially disabling conditions for bearers who have their independence, autonomy, freedom, self-image, and self-confidence affected [6–9]. Furthermore, worldwide life expectancy has risen, favoring an increase in the incidence of age-dependent diseases and the emergence of multiple chronic disorders in the same patient [10].

Even with the increasing search for new treatments, there is still a lack of pharmacological therapies able to efficiently control or interfere in the progression of these ailments [8, 11]. Additionally, currently known pharmacology profiles, in general, tend to be associated with adverse side effects that limit treatment [12, 13]. Considering this dismal panorama for the incipient treatment of the most common neurological disorders, research is underway to unveil new applications of natural products for modern drug discovery processes.

On this aspect, the efficacy of peptides and acylpolyamines isolated from arthropod venoms has attracted growing interest due to their wide range of systemic effects, including the central nervous system (CNS). In the case of the mammalian CNS, arthropod venom compounds may present either analgesic, anxiolytic, antiepileptic or neuroprotective effects, acting as inhibitors or stimulants on specific structures, such as ion channels, neurotransmitter receptors, and transporters [14–16]. Arthropod neurotoxins also function as agonists or antagonists of metabotropic and ionotropic receptors for neurotransmitters, such as the excitatory neurotransmitter glutamate [16]. In this context, this review aims to describe the main compounds found in arthropod venoms that have already been tested for the treatment of the five most prevalent neurological disorders: stroke, Alzheimer’s disease (AD), epilepsy, Parkinson’s disease (PD) and pathological anxiety.

## Review

### Cerebrovascular disease and pharmacological challenges

Cerebrovascular disease comprises stroke and other complications involving brain blood vessels. This clinical syndrome affects approximately 16.9 million individuals per year worldwide and is the second leading cause of death [17]. The World Health Organization (WHO) defined stroke as a “clinical syndrome of rapid onset of focal (or global, as in subarachnoid hemorrhage) cerebral deficit, lasting more than 24 h (unless interrupted by surgery or death), with no apparent cause other than a vascular one” [18]. However, this concept is now considered obsolete. Recently, a consensus document for an updated definition of stroke for the 21^st^ century was proposed [19]. In summary, according to Sacco et al. [19] “a CNS infarction is defined as brain, spinal cord, or retinal cell death attributable to ischemia, based on neuropathological, neuroimaging, and/or clinical evidence of permanent injury. It also broadly includes intracerebral hemorrhage and subarachnoid hemorrhage.”

Aiming to prevent or minimize brain damage resultant from ischemic cell death, several approaches have focused on potential neuroprotective treatment and/or therapeutic targets for stroke. First, considering that excessive activation of glutamate receptors is involved in brain damage following stroke, both agonist and antagonist of ionotropic glutamate receptors and their effects in exacerbating and attenuating, respectively, the post-hypoxic/ischemic outcome have been evaluated [20]. However, multiple human trials of glutamate synthetic antagonists have failed to indicate effective neuroprotection against stroke whereas side effects associated with this class of compounds have impeded clinical application [21, 22]. In this context, search for new options of non-toxic and effective antagonists has been stimulated.

Glutamate receptor antagonists have been found in arthropod venoms, mostly acylpolyamines, evaluated for alleviating brain damage caused by stroke in preclinical models [14]. Polyamine amide toxins or acylpolyamines are selective non-competitive glutamate receptor (Glu-R) antagonists able to block open channels, serving as a platform to design new drugs for the treatment of stroke in humans. For instance, the small molecule NPS-1506 (delucemine), whose design is based on argiotoxin-636 (acylpolyamine isolated from *Argiope aurantia*), blocks glutamate receptors of the NMDA-type in neurons, thus preventing excessive Ca^2+^ influx during ischemia, which produces neuroprotective effects [23]. NPS 1506 also attenuates memory dysfunction and reduces neuronal damage induced in several stroke models [21]. Unfortunately, the use of this drug as a stroke injury reducer has been discontinued in clinical stages. In 2005, the molecule, now called delucemine, was evaluated for use as an antidepressant; however, after successful outcomes in drug development phase I, research on this drug was suspended. Unexpectedly, in 2006, Johnson & Johnson Pharmaceuticals acquired intellectual property related to delucemine, and later, apparently, kept the project on hold [24].

Venom from the funnel web spider *Agelenopsis aperta* contains a mixture of atypical polyamine toxins forming a fraction termed FTX [25, 26]. FTX abolishes Ca^2+^ action, resulting in plateau potentials, and inhibits voltage-sensitive calcium channel function (VSCC) in adult cerebellar Purkinje cells (P-type channel is the predominant type of Ca^2+^ channel) [25, 27]. The active component of crude FTX, FTX-3.3, was isolated and its electrophysiological properties studied, showing that it preferentially blocks P-type VSCC, also blocking N-and L-type VSCC [28]. Considering the potential involvement of VSCC in cellular Ca^2+^loading during ischemic depolarization, these toxins, in modified form, could be useful neuroprotective agents in the case of stroke.

In relation to peptides, toxin PnTx4-3, isolated from venom of the spider *Phoneutria nigriventer*, interacts with glutamatergic neurotransmission [29–31]. Assays performed using rat brain synaptosomes revealed a dose-dependent inhibition of glutamate uptake; however, action studies in animal models for neurological disorders, focused on evaluating the neuroprotective effect have not yet been completed [30].

Two other peptides, PhTx3-3 and PhTx3-4, also isolated from *P. nigriventer* with neuroprotective action against ischemia neuronal damage, have been purified [32]. These compounds represent broad-spectrum Ca^2+^ channel blockers able to abolish both the calcium-dependent glutamate release and increase (Ca^2+^) induced by K^+^ depolarization from synaptosomes [33]. Importantly, PhTx3 decreases neuronal death and loss of neurotransmission in hippocampus CA1 tested in an *in vitro* ischemia model [34].

Acidosis has been highlighted as a common feature of ischemia, playing a critical role in brain injury. Acidosis activates Ca^2+^-permeable acid-sensing-ion channels (ASICs), causing an influx of calcium ions into neurons, thus inducing glutamate receptor-independent, Ca^2+−^dependent, neuronal injury inhibited by ASIC blockers. This calcium influx can be blocked by PcTX venom obtained from the tarantula *Psalmopoeus cambridgei*, a specific blocker of the ASIC1a subunit. In the case of focal ischemia, intracerebrovascular (ICV) injection of ASIC1a blockers protects the brain from ischemic injury and apparently does so more potently than glutamate antagonism [22].

### Alzheimer’s disease and pharmacological challenges

Alzheimer’s disease (AD) is a form of dementia characterized by the loss of cognitive function involving memory loss, deficits in comprehension and speech, and a lower capacity for recognizing objects and making plans. Dementia affects 35.6 million people worldwide, wherein AD represents 60-70 % of these cases. The risk of developing AD increases proportionally with age; thus, considering the progressive higher life expectancy, it is estimated that the frequency of cases will double by 2030 and triple by 2050, making AD an important problem of public health [35].

The main pathological hallmark found in AD patients is the presence of amyloid-β (Aβ) aggregates and neurofibrillary tangles of tau protein [36]. Misbalance between production of peptide Aβ, naturally found in neurons, and clearance causes its accumulation and aggregation [37]. In turn, tau protein, also normally found in neurons, can be hyperphosphorylated, aggregating and forming the filamentous structures that ultimately originate the tangles. Due to these neuropathological alterations, neuronal loss occurs, especially in the hippocampus and basal forebrain, as well as synaptic dysfunction and neurotransmitter deficits [38].

Therapeutic approaches remain focused on the symptomatic treatment of the disease, as there is no approved drug able to effectively modify the progression of AD in patients [39, 40]. Unfortunately, pharmacological companies have obtained ineffective results in relation to safety or efficacy in advanced clinical trials [41]. Two classes of symptomatic drugs currently in use are the acetylcholinesterase inhibitors (donepezil, galantamine and rivastigmine), which increase the availability of acetylcholine at the synaptic cleft, and the N-methyl-D-aspartic acid receptor antagonists (memantine), used to prevent glutamatergic excitotoxicity [42]. These drugs have been tested in patients with mild to moderate AD [41]. Another approach aimed at treating symptoms in patients with mild-to-moderate AD include the use of immune globulin intravenous (IGIV 10 %), a biologic agent containing human immunoglobulin IgG antibodies with anti-inflammatory and immunomodulating properties. IGIV binds to oligomeric and amyloid-β fibrils to reverse neurotoxic events in clinical and preclinical settings [43].

Due to this lack of new treatments, natural products may represent an important source of bioactive compounds with therapeutic potential for AD. For instance, acetylcholinesterase inhibitors have been isolated from natural products, especially plants [44, 45]. However, few studies have actually focused on exploring arthropod venom compounds for AD, despite their potential against other neurological disorders.

The effect of peptide Tx3-1, a selective blocker of A-type K^+^ currents (*I*_*A*_), extracted from spider venom from *P. nigriventer*, was evaluated in cognitive models in mice. Results showed the ability of the toxin to enhance both short- and long-term memory consolidation in mice tested in the novel object recognition task. Moreover, Tx3-1 restored memory of Aβ_25–35_-injected mice and exhibited higher potency to improve memory of Aβ_25–35_-injected mice when compared to control group [46].

Even though it has not been tested yet on specific models of AD, apamin, a stable octadecapeptide isolated from bee *Apis mellifera*, has been proven an important tool in learning and memory studies as it is considered a blocker of small conductance calcium-activated K^+^ channels in the CNS. This blockade may enhance the excitability of hippocampal CA1 neurons, lower threshold induction for synaptic plasticity, facilitate hippocampus-dependent memory and long-term potentiation, and enable processes apparently coupled with the post-synaptic NMDA receptor activation. Due to its neurobiological action, apamin has been proposed as a therapeutic agent that could be used to ameliorate cognitive AD symptoms [47].

### Epilepsy and pharmacological challenges

Epilepsy is characterized by a persistent predisposition to generate spontaneous epileptic seizures and by the psychological, neurobiological, social, and cognitive consequences associated with it [48, 49]. In relation to treatment, antiepileptic drug (AED) therapy aims to eliminate or reduce seizures to the maximum degree possible, thus decreasing adverse effects, helping patients in maintaining or retrieving their usual activities, and conserving a normal lifestyle [50]. Nevertheless, despite drug availability, approximately 30 % of patients with epilepsy continue to live with uncontrolled seizures. These patients are regarded as therapy-resistant or refractory to the existing AEDs [51].

In this scenario, arthropod venoms may represent an extraordinary source of bioactive molecules that act with selectivity and specificity in the mammalian CNS. This is sustained by a growing number of studies that show its true efficacy in several animal epilepsy models. Such is the case of scorpions that possess venoms with extremely specific and potent peptides that interact specifically with Na^+^, K^+^, Cl^−^ and Ca^2+^ ion channels, and also modulate GABAergic neurotransmission [52–54].

In 1989, polypeptides isolated from *Mesobuthus martensi* Karsch (BmK) venom demonstrated antiepileptic activity (BmK-AEP) in a Coriaria lactone-induced epilepsy model [55]. Later, BmK IT2, a β-like neurotoxin (BmK venom subtype) showed antiepileptic activity in pentylenetetrazole (PTZ)-induced seizures, decreased the severity of *status epilepticus* (SE), and suppressed c-Fos expression during SE induced by lithium-pilocarpine [56]. Similarly, from the venom of *M. martensi*, a sodium channel modulator (BMK AS) exhibited antiepileptic activity by inhibiting PTZ-induced seizures, and reduced the severity of the pilocarpine (PILO) seizures induced by intrahippocampal injection in rats [57]. Additionally, a novel peptide (Cll9) isolated from the venom of *Centruroides limpidus limpidus* Karsch was tested in rats. Tests showed that when this toxin is injected via ICV, it immediately induces sleep and additionally exerts marked antiepileptic action, preventing signs of penicillin-induced epileptiform activity, suggesting a potentially strong neurodepressant action in mammals [58].

During the last decades, approximately 1700 bioactive candidates have been discovered in spider venoms [24]. Some of these compounds present neuroprotective and antiepileptic effects acting on voltage-sensitive Na^+^ and Ca^2+^ channels or on glutamate receptors [52]. The observed effects are attributed to small molecules, found in spider venoms, that present the ability to recognize and antagonize mammalian receptors highly similar to invertebrate receptors, the main target of spiders [59, 60].

Neuroactive effects of polyamine toxins obtained from spiders were first recorded by Kawai *et al*. in 1982 [61], in the venom of *Nephilia clavata*. They demonstrated that JSTX blocks glutamatergic synapses in the mammalian brain. Later, Himi *et al*. [62] demonstrated that JSTX antagonized AMPA-induced seizures with no behavioral toxicity at the effective dosages. Later, JSTX-3 was synthesized and antagonized quisqualate-induced seizures; however, it did not block NMDA or kainic acid (KA)-induced convulsions, suggesting a potential activity on non-NMDA receptors in vertebrate CNS [62].

Argiotoxins identified from the venom of the spider *Argiope lobata* and other members of the Araneidae family presented neuroprotective action against KA and inhibited cell death mechanisms that involve ionotropic GluR [63–65]. Moreover, argiotoxin-636 displayed an antiepileptic effect against NMDA-induced and audiogenic seizures in mice [66]. Other polyaminic compounds also obtained from the venom of the spider *A. aperta* (AG2) exhibited antiepileptic action by blocking KA-, picrotoxin (PICRO)- and bicuculline (BIC)-induced seizures in rats [67].

Parawixin2 (FrPbAII), a toxin isolated from the venom of *Parawixia bistriata* spider, has now become a product sold by Santa Cruz Biotechnology [68, 69]. This compound selectively inhibits synaptosomal GABA and glycine reuptake in a dose-dependent manner with little or no effect on monoamine or glutamate transporters [68, 70]. Also, parawixin2 showed protection against PILO-, PICRO-, KA- and PTZ-induced seizures and PTZ-induced kindling [70, 71]. Antiepileptic activity was also identified when parawixin2 was injected into the substantia nigra pars reticulata (SNr) [72].

Similarly, parawixin10 was isolated from the same spider venom and demonstrated protection against KA, NMDA, and PTZ induced seizures in a dose-dependent manner. However, in synaptosomes from rat cerebral cortices it increased L-Glu and glycine uptake, whereas GABA uptake did not change, suggesting high-affinity mediation [73].

A neuroactive fraction obtained from spider *Scaptocosa raptorial* (SrTx1) also inhibited GABA uptake, further presenting antiepileptic effects in an acute GABAergic model of seizure induction [74]. The SrTx1.3 component from this fraction also protected against BIC-evoked seizures in a dose-dependent manner [75].

Animals submitted to intense sound stimulation were protected from tonic seizures by the administration of ω-agatoxin IVA, a peptide isolated from *A. aperta*, suggesting that it is able to antagonize NMDA receptor function [76]. Also, from the same spider, agatoxin-489 was able to block KA-induced seizures [77].

Similar to spider venom, the venom from the solitary wasp *Philanthus triangulum* contains a group of polyamine toxins called philanthotoxins (α-, β-, γ-, δ-PhTx), under investigation since the 1980s due to their remarkable activity in glutamatergic receptors [78, 79]. Furthermore, a neuroprotective and inhibitory effect on neuronal nicotinic Ach receptors has been reported for PhTx toxins and their analogues [80–82]. Despite the fact that an antiepileptic activity of these toxins has not been described in an experimental model, their selective antagonism in ionotropic glutamate receptors and possible neuroprotective effect represent at least a possibility that deserves to be further investigated [65].

As to neuroactive peptides from wasp venoms, α-pompilidotoxin (α-PMTX) was isolated from the venom of the solitary wasp *Anoplius samariensis*. α-PMTX acts mainly on presynaptic terminals by blocking the inactivation of Na^+^ channels, increasing neurotransmitter release, and enhancing synaptic activity [83]. Nonetheless, it was demonstrated that α-PMTX, when infused in cultured rat cortical neurons synchronously firing, was able to stop synchronous activity. A similar but more potent toxin, called β-pompilidotoxin, was found in the venom of *Batozonellus maculifrons* solitary wasps and acts to enhance excitatory postsynaptic potentials by reducing the inhibitory potentials in CA1 pyramidal neurons and similarly on Na^+^ channels of both central and peripheral nervous system [84–88]. Considering that slow inactivation kinetics of Na^+^ currents may thus be of functional significance for epileptogenesis (since it underlies a variety of bioelectric phenomena that culminate in increased neuronal excitability), the capacity of β-PMTX for slowing these inactivation currents raises the hypothesis that it may be a convulsive agent when tested *in vivo* [89]. So far, these studies have yet to be conducted; however, α- and β-PMTX may represent an important tool for understanding the role of such sodium currents in epileptogenesis.

As to the antiepileptic effects of the low-molecular-weight portion in wasp venoms, toxins from three social wasps from the *Polybia* genus have been described in the literature. Intracerebrovascular administration of the denatured venom from *Polybia ignobilis* and *Polybia occidentalis* were mainly effective against seizures chemically induced by BIC and KA [90, 91]. Later, the ultrafiltered venom of *Polybia paulista* was tested in a subcutaneous PTZ model, significantly increasing the latency for epileptic seizure onset [92]. Furthermore, a compound with an epileptic activity was isolated from *P. paulista* venom and subsequently denominated polybioside [93]. Rats received ICV injection of the toxin and presented tonic clonic seizures five minutes after administration, a behavior also observed with peripheric injection demonstrating this toxin’s ability to cross the blood–brain barrier [93].

The effect of the denatured venom of *Agelaia vicina* in cerebrocortical synaptosomes was described [94]; subsequently, two peptides that act on excitatory and inhibitory neurotransmissions were isolated (AvTx7 and AvTx8) [95, 96]. These works thus corroborate the necessity for more complete studies on the micromolecular fractions of wasp venoms to identify and characterize the components responsible for these notorious antiepileptic effects.

### Parkinson’s disease and pharmacological challenges

Parkinson’s disease (PD) is a progressive, neurodegenerative, and chronic pathology that affects the CNS, involving mainly the basal ganglia [97, 98]. Selective neuronal loss in the substantia nigra pars compacta (SNc) and the subsequent loss of striatal dopamine content are accepted as the pathological hallmarks for this disorder [13, 99]. Moreover, the presence of eosinophilic inclusion bodies (termed Lewy bodies and Lewy neurites, with α-synuclein being the main component) in the cytoplasm of SNc neurons as well as in other regions, and progressive mitochondrial impairment activity, are admittedly involved in the development of PD [100, 101]. Nigrostriatal pathway disruption leads to dopamine deficiency, mainly affecting motor function to cause bradykinesia, rigidity, rest tremor, and loss of postural stability, among other symptoms [13, 102, 103].

Neurodegenerative disorders, such as PD and AD, have raised great interest due to their impact on society. In the case of PD, the most common treatment strategy involves dopamine replacement with levodopa and dopamine receptor agonists [102]. Even though these treatments can provide dramatic initial improvement, their effects are often short lived, and may cause adverse motor and mental reactions, significantly limiting therapy. Moreover, neither levodopa nor other antiparkinsonian drugs are capable of arresting the progression of the disease and the deterioration of the nigrostriatal system [103]. Thus, considering these limitations, other treatment approaches are clearly needed for the development of new drugs to treat the symptoms, provide neuroprotection, and regenerate lost dopaminergic neurons.

In relation to neuroprotection for PD treatment, arthropod venoms have shown promising results. For instance, social hymenoptera possesses venom with compounds that present affinity and specificity for elements that participate in synaptic transmission in mammals [15, 104]. In this context, bee venom (BV) (also known as apitoxin) from *Apis mellifera*, when associated with acupuncture, has demonstrated the amazing capacity to interfere in disease progression in rodents with PD induced by 1-methyl-4-phenyl-1, 2, 3, 6-tetrahydropyridine (MPTP) [105]. More recently, BV associated with acupuncture also demonstrated promising results in patients with idiopathic PD, showing significant improvement on the Unified Parkinson’s Disease Rating Scale [106].

Similar results were also observed in mice treated with subcutaneous BV injections, demonstrating a potent neuroprotective effect on SNc after MPTP-induced lesion [105, 107]. BV neuroprotective effects were associated with the suppression of microglial activation and a reduction in the infiltration of CD4T cells in an MPTP mouse model for PD [108]. Under pathogenic conditions, microglia, an integrant of the innate immune system in the CNS, are rapidly activated in response to neuronal damage, significantly contributing to secondary neuronal loss [109]. *In vitro* assays revealed that BV pretreatment increased cell viability and decreased apoptosis, assessed by DNA fragmentation and caspase-3 activity in MPPT-induced cytotoxicity in SH-SY5Y human neuroblastoma cells, demonstrating BV potent anti-apoptotic effect [110]. Thus, BV suppressed neuroinflammatory responses reducing microglial activation, consequently decreasing nitric oxide expression, and preventing apoptosis via PI3K/Akt-mediated signaling pathway [107, 110].

Recently, Dantas *et al*. [111], when studying the effect of melittin on apomorphine-induced stereotypy, a component that constitutes 40 to 60 % of dry whole honeybee venom, found various biological activities, including high anti-inflammatory effect [112]. Melittin (0.1 mg/kg) and BV (0.1 mg/kg and 1.2 mg/kg) decreased the number of rotations induced by apomorphine, indicating an interaction of BV components with the dopaminergic system that might benefit the development of new BV-based strategies to treat PD. Protection from neurodegeneration was also observed by BV intraperitoneal injection, showing neuroprotection in a chronic PD model with repeated injection of toxic MPTP. Moreover, the study clearly showed that the neuroprotective effects did not simply restore TH expression in a population of diseased neurons. In the same study, authors evaluated the effect of apamin (peptide isolated from BV, detailed in section “Alzheimer’s disease and pharmacological challenges”) to restore animal motor condition. However, apamin reproduced these protective effects only partially, suggesting that other BV components enhance peptide protective action [113]. Research remains to be performed to elucidate the action mechanisms of these compounds and their potential for future clinical applications [111].

### Pathological anxiety and pharmacological challenges

Pathological anxiety corresponds to the most prevalent psychiatric disorder and according to the Diagnostic and Statistical Manual of Mental Disorders published by the American Psychiatric Association includes: generalized anxiety disorder (GAD), phobias, obsessive-compulsive disorder (OCD), post-traumatic stress disorder (PTSD), and panic disorders [114]. These disorders are characterized by excessive worrying, uneasiness, apprehension, and fear of future events as a consequence of a complex emotional response. It involves multiple neurotransmitters acting in various receptors within the limbic system and paralimbic regions of the cerebral cortex [114, 115].

Pharmacological therapy for anxiety has evolved through the years and has become more tolerable, available, and numerous. In addition to benzodiazepines, antiepileptics, antidepressants, and antipsychotics have also been used to treat anxiety disorders [115, 116]. Although in recent decades the pharmacological treatment for anxiety disorders has become increasingly more available, there is still a great need to develop new drugs with greater efficacy and tolerability, limited abuse potential, and fewer side effects [116]. In this sense, arthropod venoms may be useful for the development of novel pharmacological tools directed toward the treatment of pathological anxiety.

As previously reported, polyamine toxins belong to a class of neurotoxins widely studied in arthropods venoms. Neurological activity of parawixin2, isolated from the spider *P. bistriata*, demonstrated anxiolytic effects [68, 72]. This activity was observed in a dose-dependent manner in anxiety models of elevated plus maze and light–dark choice test after acute intrahippocampal injection of parawixin2 in rats. As previously suggested, the action mechanism proposed in this case is the blockade of GABAergic transporters, suggesting that the acute inhibition of the GABAergic transport is capable of causing anxiolytic-like behaviors due to an increase in endogenous GABAergic activity [68, 74, 117].

Some wasp venoms also showed activity in anxiety models or in assays correlated with this analysis. The AvTx8 molecule, isolated from the venom of the social wasp *A. vicina*, demonstrated its action on neuronal circuits of GABA in the substantia nigra by modulating fear responses evoked by the GABAergic blockade of the superior colliculus, which is a model used in the study of panic disorder [96].

Recently, Couto *et al*. [92] observed that the peptide fraction LMWC-Pp, obtained from *P. paulista* venom, presented anxiolytic activity in an elevated plus maze model, increasing the time spent on and the number of entries into the open arms. Yet another interesting finding is that the groups did not differ in relation to the number of entries into the closed arms of the maze. Some studies have shown that the frequency of entries into the closed arms may be an indicator of change in overall motor activity, suggesting that the compound present in the LMWC-Pp has minimal side effects [72, 118].

The scarcity of studies directly correlating neurotoxins from arthropod venoms and anxiety disorders, coupled with the great potential of some molecules elucidated for the treatment of this pathology, reveals a highly promising and still underexplored scientific area.

## Conclusions

The high social and financial impact of neurological disorders have prompted an intense search for therapeutic alternatives to the current array of drugs is urgent considering that most of these disorders have an ineffective or only symptomatic treatment. In parallel, considering that working with crude venom material involves many challenges (i.e.: high costs and bioethical issues), recent advances in the fields of bioanalytics and bioinformatics, and innovative approaches to drug discovery based on venomics and proteomics, bring hope to the discovery of new bioactive molecules capable of treating several types of diseases, including neurological disorders. Our review highlighted several promising toxins for the treatment of the top five neurological disorders. Interestingly enough, some of these toxin compounds are already being used as pharmacological tools or are being evaluated for the production of drugs for future clinical use.
